# Long-Term Regional Pathology-Detection Trends in High-Grade Cervical Lesions and Cervical Squamous Cell Carcinoma Among Women Aged 40–69 Years in Northern Norway, 1998–2025

**DOI:** 10.3390/diagnostics16172860

**Published:** 2026-09-05

**Authors:** Astrid Helmersen Aas, Elin Synnøve Mortensen, Sveinung Wergeland Sørbye

**Affiliations:** 1 Department of Medical Biology, UiT The Arctic University of Norway, 9037 Tromsø, Norway; aaa089@post.uit.no (A.H.A.); elin.mortensen@unn.no (E.S.M.); 2 Department of Clinical Pathology, University Hospital of North Norway, 9038 Tromsø, Norway

**Keywords:** cervical cancer screening, HPV testing, cervical cytology, cervical intraepithelial neoplasia, CIN2+, CIN3+, squamous cell carcinoma, pathology detection, Norway

## Abstract

**Background/Objectives:** Norway has transitioned from cytology-based to HPV-based cervical screening. We examined long-term trends in histologically confirmed cervical intraepithelial neoplasia grade 2 or worse (CIN2+), grade 3 or worse (CIN3+), and cervical squamous cell carcinoma (SCC) among women aged 40–69 years in Troms and Finnmark, a population largely ineligible for programme-based HPV vaccination. **Methods:** This retrospective, regional repeated cross-sectional study used routine pathology records from the University Hospital of North Norway from 1998 to 2025. Cervical samples obtained through primary screening, follow-up, and clinical indications were included. The most severe histological diagnosis per woman and calendar year was retained. Annual detection rates were calculated per 1000 women with at least one registered cervical sample. Temporal trends were assessed using negative binomial regression with annual endpoint counts as outcomes and the annual cervical-sampling denominator as an offset. All SCC diagnoses were included irrespective of symptoms or mode of detection. **Results:** The study comprised 350,868 annual woman-records, 3049 CIN2+, 1596 CIN3+, and 143 SCC diagnoses. CIN2+ detection increased significantly over time (rate ratio [RR] per calendar year, 1.025; 95% confidence interval [CI], 1.016–1.035; *p* < 0.001), whereas CIN3+ detection decreased (RR, 0.987; 95% CI, 0.980–0.995; *p* = 0.002). No significant temporal trend was observed for SCC (RR, 0.995; 95% CI, 0.971–1.020; *p* = 0.704). **Conclusions:** CIN2+ detection increased, CIN3+ detection decreased, and SCC showed no significant temporal trend during substantial changes in screening and diagnostic practice. The independent contributions of screening modality, screening intervals, diagnostic activity, histopathological practice, local HPV mRNA-based quality assurance, and denominator changes cannot be separated. The reported rates represent regional pathology detection relative to cervical-sampling activity rather than population incidence.

## 1. Introduction

Cervical cancer remains an important global health problem despite being largely preventable through HPV vaccination, screening, and treatment of precursor lesions [1,2]. According to the GLOBOCAN 2024 estimates, cervical cancer was the fifth most commonly diagnosed cancer among women worldwide, with approximately 604,000 new cases and 280,000 deaths in 2024 [3]. Persistent infection with oncogenic human papillomavirus (HPV) is the causal factor in almost all cervical cancers [4]. The usually prolonged interval between HPV infection, the development of high-grade precursor lesions, and invasive cervical cancer provides opportunities for prevention through vaccination and screening [5]. Cervical screening is intended to identify women with clinically relevant HPV infections and to detect high-grade cervical lesions that can be treated before progression to invasive cancer [6]. HPV testing is more sensitive than cytology for detecting high-grade cervical lesions, and a negative HPV test provides greater and longer-lasting reassurance against subsequent CIN3+ and cervical cancer [7,8]. Accordingly, when transitioning from cytology-based to HPV-based screening, the interval between screening rounds can be extended from three years after a negative cytology result to at least five years after a negative HPV test [9]. However, changes in screening modality, screening intervals, referral criteria, and diagnostic intensity may substantially affect both the number and grade distribution of lesions detected over time [9,10]. Cervical intraepithelial neoplasia grade 2 or worse (CIN2+) represents a broad measure of clinically relevant lesions that may require surveillance or treatment, whereas CIN3+ is a more stringent endpoint that more specifically reflects advanced cervical precancer and subsequent risk of invasive cancer [11,12]. Accordingly, examining both endpoints provides complementary information when evaluating changes in screening performance over time [11,12].

The organised Norwegian Cervical Cancer Screening Programme (CervicalScreen Norway) was implemented nationally in 1995 and targets women aged 25–69 years [13]. The programme initially relied on cytology at three-year intervals, with HPV testing subsequently introduced in stages for triage and follow-up [13]. At the University Hospital of North Norway (UNN), five-type HPV mRNA and 14-type HPV DNA assays were used for the triage of equivocal and low-grade cytological abnormalities [14]. Between 2016 and 2020, UNN supplemented cervical cytology with a three-type HPV E6/E7 mRNA assay targeting HPV16, HPV18, and HPV45 as a local quality-assurance measure, including the re-evaluation of HPV mRNA-positive samples initially classified as normal cytology [15]. At UNN, primary HPV DNA screening was introduced for women aged 34–69 years in January 2019 and subsequently expanded. From 1 July 2023, HPV testing became the primary screening method for all women aged 25–69 years in Norway, generally at five-year intervals [13,16].

These changes may affect histological detection in several ways. The greater sensitivity of HPV-based screening, combined with supplementary quality assurance, may increase the detection of CIN2 and enable clinically relevant lesions to be identified at an earlier stage [7,10,15]. Earlier detection and treatment of cervical precancer may, in turn, reduce subsequent progression to CIN3+ and invasive cervical cancer [9,12]. Conversely, variation in screening participation and in the annual number of women with at least one registered cervical sample may affect absolute case counts independently of changes in the underlying risk of disease [10,13]. Relating annual histological outcomes to the number of women with at least one registered cervical sample may therefore provide a more informative measure of temporal change than absolute numbers alone.

HPV vaccination has become an additional determinant of cervical precancer and cancer trends, particularly among younger women entering the screening programme [16]. Norway introduced school-based HPV vaccination in 2009 for girls born in 1997 or later, followed by a national catch-up programme during 2016–2019 for women born between 1991 and 1996 [16]. In contrast, women aged 40–69 years during the study period were born in 1985 or earlier and were therefore ineligible for Norway’s school-based and catch-up HPV vaccination programmes [16]. Trends in this age group are therefore expected to be largely independent of the direct effects of the national vaccination programmes. However, a limited indirect effect through herd protection from vaccination of younger birth cohorts cannot be excluded. The observed trends may therefore primarily reflect changes in screening participation, testing methods, diagnostic practice, and clinical follow-up, with any contribution from vaccination expected to be small [10,13,16].

Long-term real-world pathology data describing how cervical lesion detection changes during successive changes in screening practice are limited, particularly among older women. Previous studies have generally compared cytology-based and HPV-based screening within defined study populations and screening rounds. Such studies have demonstrated increased detection of CIN2+ during the first HPV-based screening round and reduced detection at subsequent rounds compared with cytology-based screening. However, these findings do not fully describe long-term patterns observed in routine clinical practice, where primary screening, follow-up of previous abnormalities, and clinically indicated cervical sampling coexist and where screening methods, referral criteria, and diagnostic practices change over time. UNN is the sole pathology provider for Troms and Finnmark and therefore captures nearly all cervical cytology, HPV testing, biopsy, and excisional pathology performed in the region [16]. This provides a unique opportunity to examine long-term real-world trends in cervical pathology detection using consistent regional laboratory records.

The present study evaluated annual numbers and detection rates of histologically confirmed CIN2+, CIN3+, and cervical squamous cell carcinoma (SCC) among women aged 40–69 years in Troms and Finnmark from 1998 to 2025. Rather than comparing predefined screening strategies or estimating their causal effects, the study describes regional real-world pathology-detection trends during 28 years of routine clinical practice spanning the transition from cytology-based to HPV-based screening. Detection rates were calculated per 1000 women with at least one registered cervical sample in the corresponding calendar year. The objective was to characterise long-term temporal trends in CIN2+, CIN3+, and SCC detection and to assess how these patterns coincided with successive changes in cervical screening and local diagnostic practice.

## 2. Materials and Methods

### 2.1. Study Design and Setting

This was a retrospective, regional, repeated cross-sectional study of temporal trends in histologically confirmed high-grade cervical lesions and cervical squamous cell carcinoma (SCC) among women aged 40–69 years residing in Troms and Finnmark, Northern Norway, between 1 January 1998 and 31 December 2025. The primary outcomes were annual detection rates of cervical intraepithelial neoplasia grade 2 or worse (CIN2+), cervical intraepithelial neoplasia grade 3 or worse (CIN3+), and SCC per 1000 women with at least one registered cervical sample.

All data were obtained from the Department of Clinical Pathology at the University Hospital of North Norway (UNN). UNN is the sole pathology provider for Troms and Finnmark and processes virtually all cervical cytology samples, HPV tests, cervical biopsies, and excisional specimens from the region. The study therefore provided near-complete regional coverage of cervical samples and histological diagnoses among women attending organised screening, undergoing follow-up of previous abnormalities, or undergoing clinically indicated cervical sampling.

The analyses assessed long-term temporal trends across the entire study period, differences across predefined calendar periods reflecting the predominant screening and triage practices, and age-stratified trends. Because changes in screening and diagnostic practice were introduced gradually and overlapped in time, the predefined periods were used descriptively and were not treated as discrete interventions or as a formal before-and-after design.

### 2.2. Cervical Screening Context

Cervical screening during the first part of the study period was predominantly cytology based. HPV testing was gradually incorporated into routine practice before the introduction of primary HPV screening. From 2006 tho 2011, women with ASC-US/LSIL cytology were triaged using the 5-type HPV E6/E7 mRNA assay PreTect HPV-Proofer (PreTect AS, Klokkarstua, Norway), targeting HPV16, HPV18, HPV31, HPV33, and HPV45. In 2012, this was replaced for triage by the 14-type cobas 4800 HPV DNA assay (Roche Diagnostics, Mannheim, Germany) [14].

In addition to testing performed according to national screening guidelines, from 2016 to 2020, UNN routinely used the 3-type HPV E6/E7 mRNA assay PreTect SEE (PreTect AS), targeting HPV16, HPV18, and HPV45, as an adjunct to cervical cytology for local quality assurance. This included testing of women with normal cytology to improve risk stratification and reduce the risk of cervical cancer after a negative cytological result [15].

Primary HPV DNA screening was introduced gradually at UNN using the cobas 4800 assay. From January 2019, women aged 34–69 years were randomised between cytology-based and HPV-based primary screening. HPV DNA testing became the primary screening method for all women aged 34–69 years in July 2021, was extended to women aged 30–69 years in January 2023, and was implemented for all women aged 25–69 years in July 2023. During primary HPV screening, women with a positive HPV DNA result were triaged using liquid-based cytology together with the genotype-specific 7-type HPV E6/E7 mRNA assay PreTect HPV-Proofer’7 (PreTect AS), targeting HPV16, HPV18, HPV31, HPV33, HPV45, HPV52, and HPV58. In 2025, the cobas 4800 platform was replaced by the cobas 6800 HPV DNA assay (Roche Diagnostics) for primary HPV screening.

The study population was selected to minimise the direct influence of the national HPV vaccination programmes. Women aged 40–69 years during 1998–2025 were born in 1985 or earlier and were therefore not eligible for either the school-based vaccination programme offered to women born in 1997 or later or the catch-up programme offered to women born during 1991–1996. Opportunistic HPV vaccination outside the national programmes could not be assessed; however, such vaccination is expected to have been uncommon in these older birth cohorts and therefore unlikely to have materially influenced the observed trends.

### 2.3. Data Source and Study Population

Data were extracted from the SymPathy laboratory information system (FlexLab/SymPathy v5.14.4.2; Tietoevry, Espoo, Finland), which was used by the Department of Clinical Pathology at UNN throughout the study period. SymPathy records cervical cytology, HPV testing, and histopathology findings together with patient age, residential information, sampling date, specimen type, anatomical site, and final coded diagnosis. Histological diagnoses were coded according to the Norwegian Pathology Code System (NORPAT), the national coding system used by pathology laboratories and for reporting pathology information to the Cancer Registry of Norway [17].

Women were eligible for inclusion in each calendar year if they were aged 40–69 years at the time of sampling, registered as residents of Troms or Finnmark, and had an eligible cervical sample or cervical histological specimen. Residence was determined using the address or postal code recorded on the laboratory requisition. Samples processed outside UNN were not captured in the dataset. Vaginal samples were not included. Hysterectomy status was not available in the pathology dataset. Records with missing or invalid patient identifiers, age, calendar year, or residence information were excluded from the relevant analysis, and no data were imputed.

A person-specific identifier was used to de-duplicate records within each calendar year. A woman could contribute one annual cervical-sampling record and one annual record for each applicable histological endpoint. The same woman could contribute data in more than one calendar year while remaining within the eligible age range. Consequently, totals across the study period represent annual woman-records rather than the number of distinct women followed throughout the entire period.

### 2.4. Cervical-Sampling Denominator

A cervical sample was defined as a specimen collected from the cervix and analysed by cytology, HPV testing, or both. Vaginal samples were excluded. The annual denominator was defined as the number of unique women aged 40–69 years with at least one registered cervical sample during the corresponding calendar year. Multiple samples, diagnostic codes, HPV genotype results, or follow-up tests from the same woman in the same year were reduced to one annual cervical-sampling record.

The denominator included samples obtained through organised screening, follow-up of previous abnormalities, and clinical indications. It therefore represents annual cervical-sampling activity rather than a population denominator or a cohort restricted to routine primary screening. Hysterectomy status was not available; however, women without a cervix who did not have a cervical sample did not contribute to the denominator, and vaginal samples were not included. The overall denominator was calculated as the sum of the annual woman-records and should not be interpreted as the number of distinct women examined during the entire study period.

### 2.5. Histological Outcomes and Case Classification

All cervical histological specimens registered between 1 January 1998 and 31 December 2025 were assessed. The material included cervical biopsies, endocervical curettage specimens, loop electrosurgical excision procedure/large loop excision of the transformation zone specimens (LEEP/LLETZ), other conisation specimens, and hysterectomy specimens containing cervical dysplasia or malignancy.

Histological outcomes were identified using the following NORPAT codes [17]:

• M74007: cervical intraepithelial neoplasia grade 2;
• M80702: cervical intraepithelial neoplasia grade 3;
• M81402: adenocarcinoma in situ; and
• M80703: invasive squamous cell carcinoma.

CIN2+ was defined as CIN2, CIN3, adenocarcinoma in situ (ACIS), or SCC. CIN3+ was defined as CIN3, ACIS, or SCC. Benign, reactive, and low-grade histological findings, including CIN1, were not included in the outcome numerators.

If several histological specimens or diagnoses were registered for the same woman during the same calendar year, only the most severe diagnosis was retained according to the following hierarchy:

SCC > ACIS > CIN3 > CIN2.

The endpoints were nested; a woman classified with SCC contributed to the annual CIN2+, CIN3+, and SCC counts, whereas a woman classified with CIN3 or ACIS contributed to the CIN2+ and CIN3+ counts. A woman with an eligible histological diagnosis in a subsequent calendar year could contribute to that year’s analysis. The outcomes therefore represent annual detection rather than first-ever or lifetime diagnoses.

All SCC diagnoses were included irrespective of symptoms, screening history, or mode of detection. No distinction was made between SCC detected through screening and SCC diagnosed following symptoms or clinical findings.

### 2.6. Detection Rates and Statistical Analysis

Annual numbers of CIN2+, CIN3+, and SCC were presented as absolute counts and as detection rates per 1000 women with at least one registered cervical sample. For each endpoint and calendar year, the detection rate was calculated by dividing the number of eligible histological diagnoses by the number of unique women with at least one registered cervical sample during that year and multiplying the result by 1000. The annual number of unique women with at least one registered cervical biopsy was also calculated as a descriptive measure of diagnostic activity.

The rates should be interpreted as annual regional pathology-detection rates scaled to the volume of registered cervical sampling rather than as population incidence rates or risks within a closed cohort. All eligible SCC diagnoses were included in the numerator, including cancers diagnosed following symptoms, regardless of whether the diagnostic episode originated from screening.

**Figure 1:**
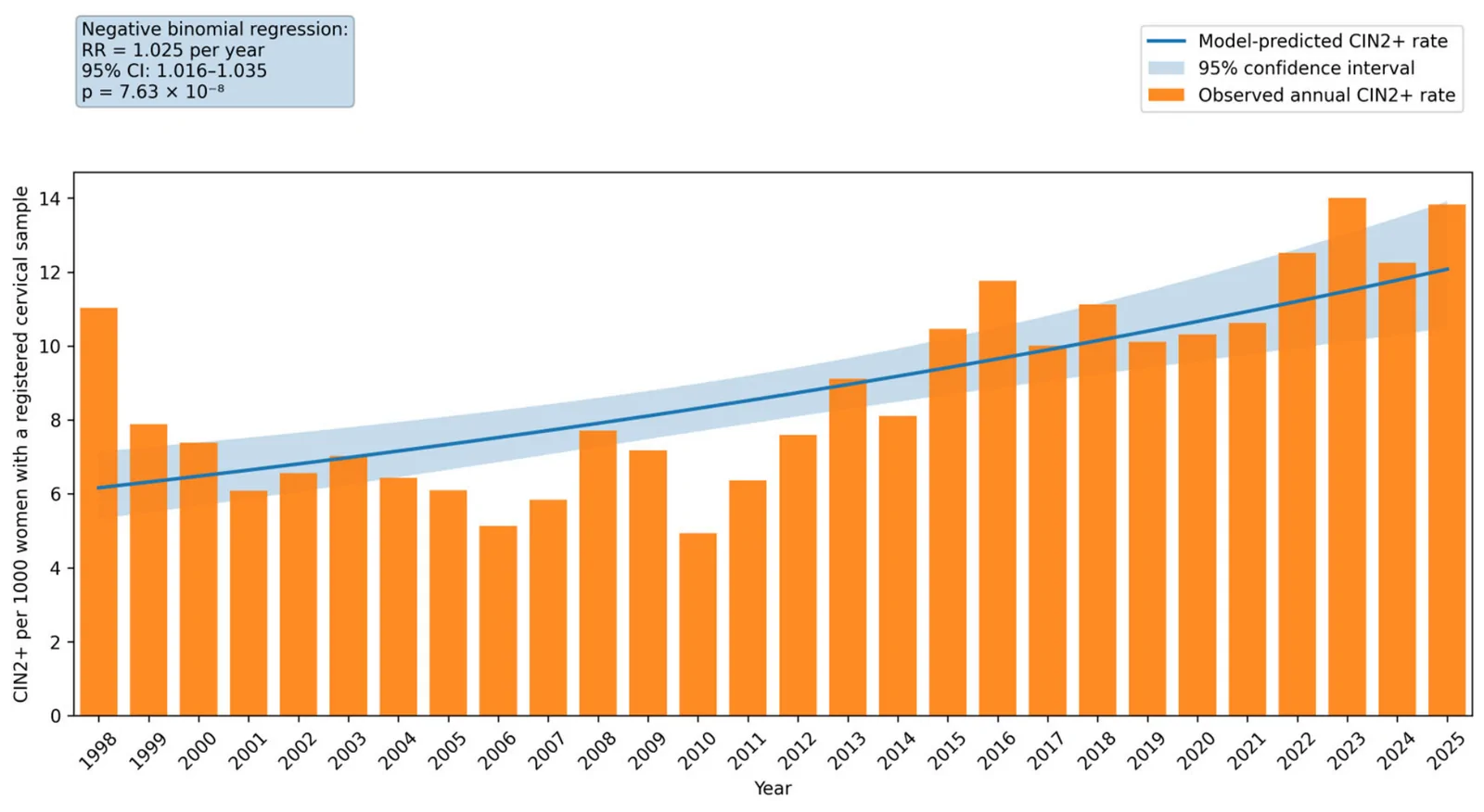
Annual CIN2+ detection rate among women aged 40–69 years in Troms and Finnmark, 1998–2025. Bars represent the observed annual CIN2+ detection rates per 1000 women with at least one registered cervical sample. The solid line represents model-predicted detection rates from negative binomial regression, with the shaded area indicating the corresponding 95% confidence interval. The model used the annual number of CIN2+ diagnoses as the outcome and the logarithm of the annual number of women with at least one registered cervical sample as an offset. The CIN2+ detection rate increased significantly over time (rate ratio [RR] per calendar year, 1.025; 95% CI, 1.016–1.035; *p* < 0.001). CIN2+ includes cervical intraepithelial neoplasia grades 2 and 3, adenocarcinoma in situ, and squamous cell carcinoma. CI, confidence interval; RR, rate ratio.

**Figure 2:**
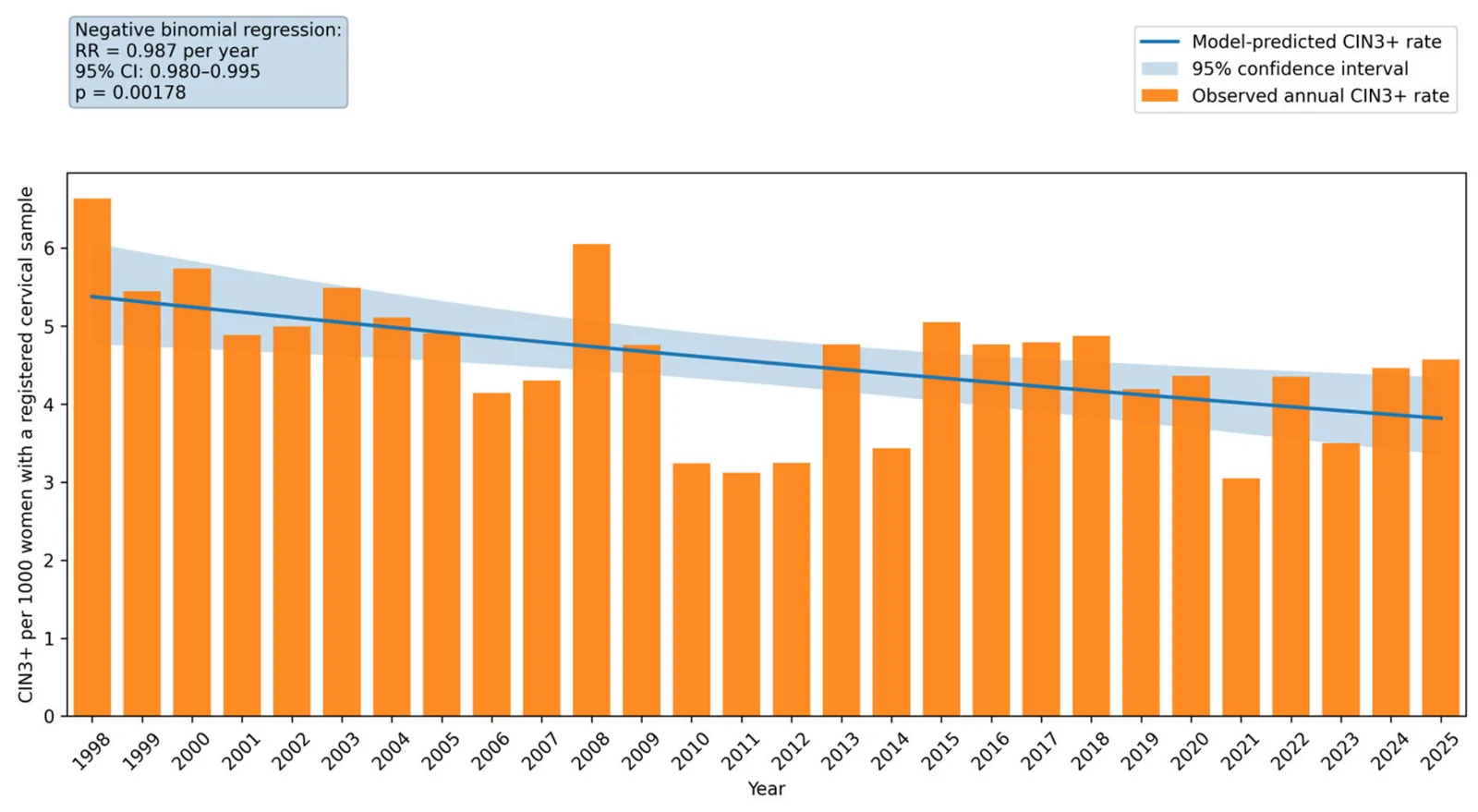
Annual CIN3+ detection rate among women aged 40–69 years in Troms and Finnmark, 1998–2025. Bars represent the observed annual CIN3+ detection rates per 1000 women with at least one registered cervical sample. The solid line represents model-predicted detection rates from negative binomial regression, with the shaded area indicating the corresponding 95% confidence interval. The model used the annual number of CIN3+ diagnoses as the outcome and the logarithm of the annual number of women with at least one registered cervical sample as an offset. The CIN3+ detection rate decreased significantly over time (rate ratio [RR] per calendar year, 0.987; 95% CI, 0.980–0.995; *p* = 0.002). CIN3+ includes cervical intraepithelial neoplasia grade 3, adenocarcinoma in situ, and squamous cell carcinoma. CI, confidence interval; RR, rate ratio.

**Figure 3:**
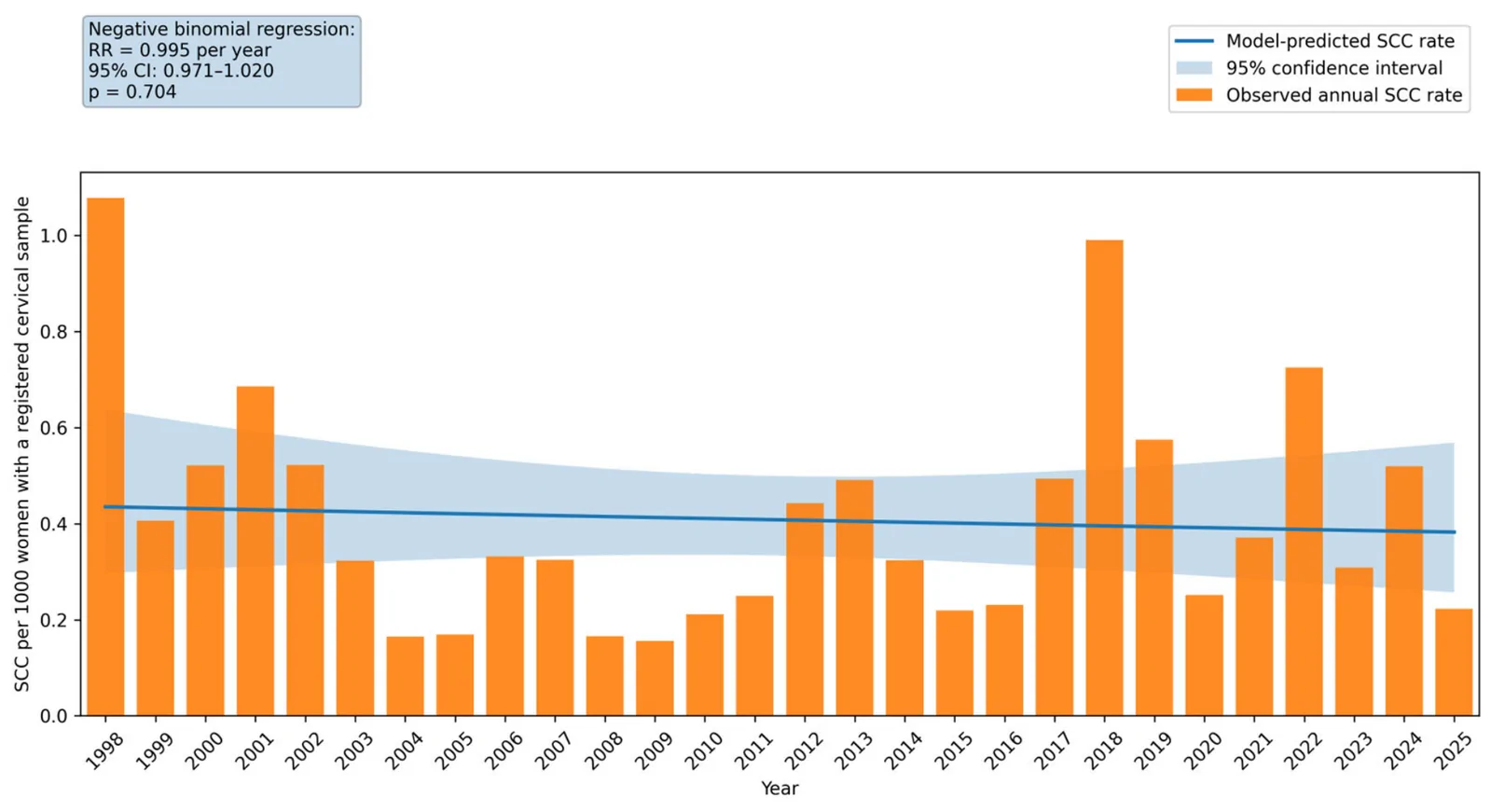
Annual cervical squamous cell carcinoma detection rate among women aged 40–69 years in Troms and Finnmark, 1998–2025. Bars represent the observed annual squamous cell carcinoma (SCC) detection rates per 1000 women with at least one registered cervical sample. The solid line represents model-predicted detection rates from negative binomial regression, with the shaded area indicating the corresponding 95% confidence interval. The model used the annual number of SCC diagnoses as the outcome and the logarithm of the annual number of women with at least one registered cervical sample as an offset. No significant temporal trend was observed (rate ratio [RR] per calendar year, 0.995; 95% CI, 0.971–1.020; *p* = 0.704). All SCC diagnoses were included irrespective of symptoms or mode of detection. CI, confidence interval; RR, rate ratio.

Long-term temporal trends in CIN2+, CIN3+, and SCC detection were assessed using negative binomial regression to account for count outcomes, varying annual denominators, and potential overdispersion. For each endpoint, the annual number of diagnoses was entered as the dependent variable, calendar year as a continuous independent variable, and the logarithm of the annual number of women with at least one registered cervical sample as an offset. Results were expressed as rate ratios (RRs) per calendar year with 95% confidence intervals and two-sided *p*-values. Model-predicted detection rates and corresponding 95% confidence intervals were used to illustrate the temporal trends in Figure 1, Figure 2 and Figure 3.

To describe changes across major phases of screening practice, the study period was also divided into five predefined calendar periods: 1998–2005, when screening was based predominantly on cytology alone; 2006–2011, when cytology was combined with 5-type HPV mRNA triage of ASC-US/LSIL; 2012–2015, when 14-type HPV DNA testing was used for ASC-US/LSIL triage; 2016–2020, when cytology was supplemented by 3-type HPV mRNA co-testing as a local quality-assurance strategy; and 2021–2025, when primary HPV DNA screening became the predominant screening strategy. These periods represent the predominant screening and triage practices and should not be interpreted as discrete interventions, as several changes were introduced gradually and overlapped in time.

Differences in CIN2+, CIN3+, and SCC detection across these periods were assessed using negative binomial regression, with the annual number of endpoint diagnoses as the dependent variable and the logarithm of the annual number of women with at least one registered cervical sample as an offset. Screening period was entered as a categorical variable. Overall differences between periods were assessed using likelihood-ratio tests. Exploratory comparisons between adjacent screening periods were expressed as RRs with 95% confidence intervals, with *p*-values adjusted for multiple comparisons using the Holm method.

To examine whether the overall temporal patterns could be explained by changes in the age distribution of women undergoing cervical sampling, additional analyses were performed separately for women aged 40–49, 50–59, and 60–69 years. Annual age-specific detection rates were calculated using the corresponding number of women with at least one registered cervical sample in each age group as the denominator. Separate negative binomial regression models were fitted for CIN2+ and CIN3+ within each age stratum, using annual endpoint counts as the dependent variable, calendar year as a continuous independent variable, and the logarithm of the corresponding age-specific annual denominator as an offset. Age-stratified SCC analyses were not performed because of the small number of cases and sparse age-specific annual counts.

The primary analyses were not age-standardised, and no adjustment was made for screening indication, previous cervical disease or treatment, HPV status, hysterectomy status, socioeconomic characteristics, migration, or other potential confounders. The analyses were therefore descriptive and were not intended to establish causal effects of individual screening methods, quality-assurance measures, or changes in local diagnostic practice. A two-sided *p*-value < 0.05 was considered statistically significant.

Data management, harmonisation, and de-duplication were performed using IBM SPSS Statistics version 32.0.0.0 (IBM Corp., Armonk, NY, USA). Statistical analyses and figures were generated using Python version 3.12.13 (Python Software Foundation, Beaverton, OR, USA). Rates displayed in tables and figures were rounded for presentation, whereas statistical analyses were based on the underlying unrounded annual data.

### 2.7. Ethics

The study was based on retrospective analysis of routinely collected pathology data. No patient contact, intervention, or collection of new biological specimens was undertaken. Person-specific identifiers were used solely for record linkage and de-duplication within the secure data environment and were not retained in the aggregated analytical outputs.

The Regional Committee for Medical and Health Research Ethics, Northern Norway (REK Nord), assessed the project as a quality-assurance project outside the scope of the Norwegian Health Research Act (reference number 203384). Formal research ethics approval and individual informed consent were therefore not required.

## 3. Results

### 3.1. Study Population and Annual Histological Outcomes

During 1998–2025, 350,868 annual woman-records with at least one registered cervical sample were recorded among women aged 40–69 years. Of these, 31,881 annual woman-records included at least one cervical biopsy, corresponding to 907–1500 women with a biopsy per calendar year. After retaining the most severe histological diagnosis per woman in each calendar year, 3049 CIN2+, 1596 CIN3+, and 143 SCC diagnoses were identified. The annual numbers ranged from 62 to 153 for CIN2+, from 34 to 80 for CIN3+, and from 2 to 13 for SCC (Table 1).

### 3.2. Cervical-Sampling Activity and Annual Detection Rates

The annual numbers of women with at least one registered cervical sample, the corresponding cervical biopsy activity, and the detection rates are presented in Table 2. Annual cervical-sampling activity ranged from 8964 women in 2025 to 16,036 women in 2011. The proportion of sampled women with at least one registered cervical biopsy varied substantially over time, ranging from 65.5 per 1000 in 2011 to 130.1 per 1000 in 2025. Across the entire study period, 350,868 annual woman-records were included, of which 31,881 included at least one cervical biopsy, corresponding to an overall biopsy rate of 90.9 per 1000. Overall detection rates were 8.69 per 1000 for CIN2+, 4.55 per 1000 for CIN3+, and 0.41 per 1000 for SCC.

### 3.3. Temporal Trend in CIN2+ Detection

The annual CIN2+ detection rate varied from 4.93 per 1000 women with a registered cervical sample in 2010 to 14.00 per 1000 in 2023. Except for the relatively high rate observed in 1998, rates generally remained below 8 per 1000 until 2012, followed by a sustained increase from 2013 onwards. Negative binomial regression, using the annual number of CIN2+ diagnoses as the outcome and the annual number of women with at least one registered cervical sample as an offset, confirmed a significant upward temporal trend. The CIN2+ detection rate increased by an estimated 2.5% per calendar year (rate ratio [RR], 1.025; 95% CI, 1.016–1.035; *p* < 0.001) (Figure 1).

### 3.4. Temporal Trend in CIN3+ Detection

The annual CIN3+ detection rate varied from 3.05 per 1000 women with a registered cervical sample in 2021 to 6.63 per 1000 in 1998. Despite year-to-year variation, the rates showed an overall decline across the study period. Negative binomial regression, using the annual number of CIN3+ diagnoses as the outcome and the annual number of women with at least one registered cervical sample as an offset, confirmed a significant downward temporal trend. The CIN3+ detection rate decreased by an estimated 1.3% per calendar year (rate ratio [RR], 0.987; 95% CI, 0.980 to 0.995; *p* = 0.002) (Figure 2).

### 3.5. Temporal Trend in Cervical SCC Detection

The annual SCC detection rate varied from 0.16 per 1000 women with a registered cervical sample in 2004 and 2009 to 1.08 per 1000 in 1998, with marked year-to-year variation. Negative binomial regression, using the annual number of SCC diagnoses as the outcome and the annual number of women with at least one registered cervical sample as an offset, showed no significant temporal trend across the study period (rate ratio [RR], 0.995 per calendar year; 95% CI, 0.971 to 1.020; *p* = 0.704) (Figure 3). Given the small number of SCC diagnoses (143 cases in total, with 2–13 cases per year), this estimate should be interpreted cautiously.

### 3.6. Detection Rates Across Screening Periods

When the study period was grouped into five predefined calendar periods reflecting the predominant screening and triage practices, the CIN2+ detection rate decreased 1 per 1000 women with a registered cervical sample in 1998–2005 to 6.18 per 1000 in 2006–2011, followed by successive increases to 8.80 in 2012–2015, 10.67 in 2016–2020, and 12.48 per 1000 in 2021–2025 (Table 3). In contrast, the CIN3+ detection rate was highest in 1998–2005 (5.40 per 1000) and remained lower in the subsequent periods, reaching 3.92 per 1000 in 2021–2025. SCC detection showed no consistent period-specific pattern, with rates ranging from 0.24 to 0.51 per 1000.

Negative binomial regression using annual endpoint counts and the annual number of women with a registered cervical sample as an offset showed significant differences across calendar periods for CIN2+ (likelihood-ratio *p* < 0.001) and CIN3+ (*p* = 0.010), but not for SCC (*p* = 0.124). In exploratory comparisons between adjacent periods with Holm adjustment for multiple testing, CIN2+ detection increased significantly from 2006–2011 to 2012–2015 (rate ratio [RR], 1.42; 95% CI, 1.19–1.70; adjusted *p* < 0.001), whereas CIN3+ detection decreased significantly from 1998–2005 to 2006–2011 (RR, 0.78; 95% CI, 0.66–0.92; adjusted *p* = 0.010). No other adjacent-period comparison remained statistically significant after adjustment for multiple testing.

### 3.7. Annual Numbers of Women with a Registered Cervical Sample by Age Group

The annual sampling denominator was also examined separately for women aged 40–49, 50–59, and 60–69 years (Table 4). Across the entire study period, 142,729 annual woman-records were contributed by women aged 40–49 years, 122,829 by women aged 50–59 years, and 85,310 by women aged 60–69 years. The annual number of women with at least one registered cervical sample varied substantially over time within each age group. The overall annual denominator peaked at 16,036 women in 2011 and declined during the final years of the study to 9716 in 2023, 9631 in 2024, and 8964 in 2025. This decline was observed across all three age strata and provides the age-specific denominators used for the CIN2+ and CIN3+ detection rates presented in Table 5.

### 3.8. Age-Stratified CIN2+ and CIN3+ Detection Trends

Age-stratified analyses showed increasing CIN2+ detection in all three age groups (Table 5). Negative binomial regression, using annual endpoint counts and the corresponding age-specific number of women with at least one registered cervical sample as an offset, showed the strongest increase among women aged 50–59 years (rate ratio [RR] per calendar year, 1.031; 95% CI, 1.015–1.048; *p* < 0.001), followed by women aged 40–49 years (RR, 1.030; 95% CI, 1.023–1.037; *p* < 0.001) and 60–69 years (RR, 1.019; 95% CI, 1.003–1.036; *p* = 0.022). Thus, CIN2+ detection increased significantly over time within each age stratum.

For CIN3+, the estimated temporal trend was negative in all three age groups. A significant decline was observed among women aged 40–49 years (RR per calendar year, 0.991; 95% CI, 0.983–0.999; *p* = 0.028). The estimated trends were also negative among women aged 50–59 years (RR, 0.992; 95% CI, 0.976–1.009; *p* = 0.359) and 60–69 years (RR, 0.983; 95% CI, 0.965–1.001; *p* = 0.067), but did not reach statistical significance. Overall, the age-stratified analyses showed that the increase in CIN2+ detection was present across all age groups, while CIN3+ detection showed a generally downward direction, supporting that the principal temporal patterns were not solely attributable to changes in the age distribution of women undergoing cervical sampling.

## 4. Discussion

### 4.1. Principal Findings

This regional study examined 28 years of cervical screening and histopathology data among women aged 40–69 years, comprising 350,868 annual woman-records, 3049 CIN2+, 1596 CIN3+, and 143 cervical squamous cell carcinoma (SCC) diagnoses. Three different temporal patterns were observed. The annual CIN2+ detection rate increased significantly, the CIN3+ detection rate declined significantly, and the SCC detection rate showed no significant temporal trend. Because the birth cohorts included in this age group were not eligible for programme-based HPV vaccination, the findings are more likely to reflect changes in cervical-sampling activity, testing methods, diagnostic practice, and clinical management than a direct effect of HPV vaccination.

The divergent trends in CIN2+ and CIN3+ are potentially important. An increasing CIN2+ detection rate accompanied by a declining CIN3+ rate is compatible with earlier identification and treatment of clinically relevant cervical lesions, leaving fewer lesions to progress to more advanced precancer [18]. However, the repeated cross-sectional design does not establish that women diagnosed and treated for CIN2 would otherwise have progressed to CIN3. The observed patterns should therefore be interpreted as temporal associations rather than evidence of a causal effect of any individual screening intervention. Age-stratified analyses showed that the increase in CIN2+ was present in all three 10-year age groups, while CIN3+ trends were negative across all age strata.

### 4.2. Diverging Trends in CIN2+ and CIN3+

The CIN2+ detection rate increased significantly over time, corresponding to an estimated 2.5% increase per calendar year (rate ratio [RR], 1.025; 95% CI, 1.016–1.035; *p* < 0.001). Rates were generally below 8 per 1000 women with at least one registered cervical sample between 1999 and 2012 but increased substantially from 2013 onwards. Several mechanisms may have contributed to this pattern. HPV-based screening is more sensitive than cytology for detecting CIN2+ and CIN3+ and may identify clinically relevant lesions earlier in the disease course [7,18]. Increased HPV testing, more intensive follow-up of HPV-positive women, and changes in referral thresholds may increase the detection of CIN2 [10]. Changes in referral and biopsy activity may also alter the histological case mix towards increased ascertainment of lower-grade high-grade lesions [19]. Because CIN2+ includes CIN2 as well as all lesions included in CIN3+, the combination of increasing CIN2+ detection and decreasing CIN3+ detection indicates that the increase in the broader composite endpoint was predominantly attributable to increased detection of CIN2 rather than CIN3+. Thus, the grade distribution of detected high-grade cervical lesions changed over time.

The age-stratified analyses support the robustness of the overall temporal patterns. CIN2+ detection increased significantly within each of the three age groups. The estimated annual increase was similar among women aged 40–49 and 50–59 years and somewhat smaller among women aged 60–69 years. Thus, the overall increase in CIN2+ cannot be explained solely by changes in the age distribution of women undergoing cervical sampling. For CIN3+, the estimated temporal trend was negative in all three age groups, but reached statistical significance only among women aged 40–49 years. The declines among women aged 50–59 and 60–69 years did not reach statistical significance. These findings indicate that the overall divergence between increasing CIN2+ and decreasing CIN3+ detection was broadly present across age strata, although the strength and statistical precision of the CIN3+ trends differed between age groups.

CIN2 is a biologically and diagnostically heterogeneous category. Some lesions show biomarker profiles consistent with productive HPV infection and may regress, whereas others show features of transforming infection and a greater presumed risk of progression [20,21]. Histological grading of cervical intraepithelial neoplasia is subject to interobserver variability, particularly around the CIN2 threshold, while biomarker-assisted assessment using p16 may improve diagnostic accuracy and reproducibility in selected cases [22,23]. Changes in pathology staffing and diagnostic practice over a 28-year period may therefore have contributed to temporal variation. Although the group of pathologists evaluating cervical specimens at UNN has been relatively stable, some changes in staffing inevitably occurred. A substantial proportion of cervical biopsies during the last 10–15 years, encompassing the period with the greatest changes in screening technology and the largest increase in CIN2+ detection, were nevertheless evaluated by the same group of pathologists. In addition, p16 immunohistochemistry has been used at UNN as an adjunct in diagnostically challenging cervical biopsies since approximately 2012. Its introduction may have influenced classification around the CIN2 threshold and contributed to increased CIN2 detection, although p16 is not primarily a discriminator between CIN2 and CIN3. Histological reporting at UNN continues to distinguish CIN2 from CIN3 because this distinction remains clinically relevant, particularly for conservative management of CIN2 in younger women. In the present study population aged 40–69 years, however, both CIN2 and CIN3 would generally lead to excisional treatment.

The annual CIN3+ detection rate decreased significantly over time, corresponding to an estimated 1.3% decrease per calendar year (rate ratio [RR], 0.987; 95% CI, 0.980–0.995; *p* = 0.002). CIN3+ is a more reproducible histopathological endpoint and a more specific marker of advanced cervical precancer than CIN2+, whereas CIN2 comprises a heterogeneous group of lesions at a diagnostically less reproducible treatment threshold [23]. The decline in CIN3+ despite increasing CIN2+ detection is compatible with a shift towards detection of lesions at an earlier stage, but increased ascertainment of biologically heterogeneous and potentially regressive CIN2 lesions may also have contributed. More sensitive screening may therefore increase treatment of CIN2 lesions that might otherwise have regressed without intervention. A similar pattern was observed in the English primary HPV screening pilot, in which increased detection of CIN2+ and CIN3+ during the initial HPV-based screening round was followed by substantially lower CIN3+ detection at the subsequent incidence screen [18]. The observational design of the present study does not establish that earlier detection caused the decline in CIN3+ or that the additional CIN2 lesions detected would otherwise have progressed. Other possible explanations include changes in the age distribution of women undergoing cervical sampling, screening intervals, referral and biopsy practices, histopathological classification, use of ancillary biomarkers, and changes in the composition of the sampled population.

The transition from three-year cytology screening to five-year HPV-based screening also complicates the interpretation of detection rates per woman with a registered cervical sample. Extending the recommended interval reduces the expected average annual sampling volume and may produce substantial year-to-year fluctuations during implementation [24]. Moreover, each screening episode represents a longer period since the preceding routine test, during which detectable lesions may develop or persist. Consequently, detection rates per woman with a registered cervical sample may be influenced by screening interval and timing, in addition to test sensitivity, referral practices, and underlying disease occurrence, and should not be interpreted as directly comparable annual incidence rates across screening strategies. The declining number of women with at least one registered cervical sample during the final years of the study therefore contributed to the higher calculated CIN2+ rates per 1000 women with a registered cervical sample, although the absolute numbers of CIN2+ diagnoses changed less markedly (Table 1 and Table 2). Thus, part of the increase in the late-period detection rates reflects a change in the denominator rather than a corresponding increase in absolute case numbers. Detection rates per woman with a registered cervical sample are useful for relating histological outcomes to annual cervical-sampling activity but should not be interpreted as population incidence rates. They remain influenced by screening intervals, attendance patterns, and the characteristics of women undergoing cervical sampling in each calendar year [24].

### 4.3. Changes in Screening Practice and HPV mRNA-Based Quality Assurance

Screening practice changed substantially during the study period. Cytology was the primary screening method during the earlier years, whereas HPV testing was introduced progressively for the triage and follow-up of equivocal and low-grade cytological abnormalities [13,14]. From 2016 to 2020, UNN used an HPV E6/E7 mRNA assay detecting HPV16, HPV18, and HPV45 as an adjunctive quality-assurance test in women with normal cytology [15]. At UNN, primary HPV DNA screening was introduced for women aged 34–69 years in 2019. From 1 July 2023, primary HPV screening was extended nationally to all women aged 25–69 years, generally at five-year intervals [13,25].

The period-specific analyses further illustrate that the temporal patterns were not uniform across the 28-year study period. CIN2+ detection was lowest during 2006–2011 and increased across each of the three subsequent periods, reaching its highest level during 2021–2025. In contrast, CIN3+ detection was highest during 1998–2005 and remained lower thereafter. However, only selected adjacent-period comparisons remained statistically significant after adjustment for multiple testing. Because the predefined periods encompass gradual and overlapping changes in screening, triage, and diagnostic practice, these differences should not be interpreted as effects of individual screening interventions.

The local mRNA-based quality-assurance strategy may have contributed to increased ascertainment of lesions among women whose cytology was interpreted as normal. HPV E6/E7 mRNA testing may provide information more closely related to transforming infection than HPV DNA positivity alone [14,15]. Previous Norwegian studies have shown that HPV16/18/45 mRNA positivity among women with normal cytology identifies a relatively small subgroup with substantially increased subsequent risk of CIN2+, CIN3+, and cervical cancer [15,26,27]. These findings provide a biologically and clinically plausible rationale for the use of targeted mRNA testing as an adjunct to cytology.

However, the present study did not link individual mRNA results to subsequent histological outcomes, and the period of mRNA-based quality assurance overlapped with other changes in screening, follow-up, and diagnostic practice. Its independent contribution to the observed temporal trends cannot therefore be quantified, and any possible contribution should be regarded as hypothesis-generating rather than as evidence of a causal effect.

The findings should not be interpreted as challenging the role of HPV DNA testing as the primary screening method. Current Norwegian guidelines recommend validated primary HPV assays detecting 12–14 oncogenic HPV types, combined with genotype-based risk stratification and structured follow-up of HPV-positive women [13,25]. However, evidence suggests that a more restricted group of the most carcinogenic HPV types accounts for the large majority of cervical cancers [28,29], consistent with the WHO target product profile for HPV screening tests [30] and data from Sweden and other Nordic countries [31,32].

At UNN, primary HPV DNA screening is complemented by cytology and the genotype-specific 7-type HPV E6/E7 mRNA assay PreTect HPV-Proofer’7, targeting HPV16, HPV18, HPV31, HPV33, HPV45, HPV52, and HPV58, for risk stratification of HPV DNA-positive women [33]. The mRNA assay is used as a triage test rather than as a replacement for primary HPV DNA screening, and women positive for HPV DNA types not targeted by the assay remain under structured follow-up. Genotype-specific HPV E6/E7 mRNA testing may therefore complement primary HPV DNA screening by concentrating CIN2+ and CIN3+ risk within a smaller test-positive subgroup while maintaining surveillance of other HPV DNA-positive women [14,15,31,32,33].

### 4.4. No Significant Temporal Trend in SCC Detection

No significant temporal trend was observed for cervical SCC. The estimated annual change was close to zero, and only 2–13 cases were diagnosed each year. This limited number of cases resulted in marked year-to-year variation and low statistical power to detect a modest change in cancer occurrence. The absence of a significant temporal trend in SCC detection should therefore not be interpreted as evidence that changes in screening had no effect.

Progression from persistent HPV infection through high-grade precursor lesions to invasive cervical cancer generally occurs over many years [5,12]. Consequently, changes in the detection and treatment of precursor lesions may not be reflected immediately in cancer rates. Primary HPV screening was introduced mainly during the latter part of the study period and was extended nationally to all eligible age groups only in July 2023 [13,25]. Follow-up after full implementation was therefore limited to approximately 2.5 years, which is insufficient to assess its full effect on cervical SCC among women aged 40–69 years. The Department of Clinical Pathology at UNN was established in 1972, and cervical cytology formed an important part of its diagnostic activities from the outset. Norway subsequently established a national organised cervical screening programme for women aged 25–69 years in 1995 [13]. Thus, when the present study began in 1998, cytology-based screening had already been practised regionally for several decades, although the national programme had been in place for only three years. The absence of a significant temporal trend in SCC detection during 1998–2025 may therefore reflect the residual cancer burden following long-standing cytology-based screening rather than an absence of screening effectiveness. The relevant counterfactual—the number of cancers that would have occurred without screening—cannot be estimated from the present data.

Previous Nordic studies have demonstrated substantial reductions in cervical cancer following organised screening. Modelling of incidence trends in Denmark, Finland, Norway, and Sweden estimated that screening prevented 41–49% of the cervical cancers that would otherwise have occurred between 1961 and 2010 [34]. A nationwide Swedish cohort study showed that cytological screening at ages 61–65 years was associated with a significantly lower subsequent risk of cervical cancer, particularly among women who had not been screened or had abnormal screening results during their 50s [35]. More recent Swedish audit data identified non-participation as the most important screening-related risk category, accounting for 31% of cervical cancer cases in 2022 [36]. Consistent with these findings, a nationwide Norwegian study found that approximately half of fatal cervical cancers occurred among screening non-attenders. Among women diagnosed at ages 25–69 years, approximately 75% had no registered cytology during the preceding 3.5 years [37].

All SCC diagnoses were included irrespective of symptoms or mode of detection. This approach avoided potential misclassification arising from separation of screen-detected and symptom-detected cancers but meant that the SCC numerator included cancers diagnosed in women who did not necessarily have a registered cervical sample during the corresponding year. Consequently, the SCC rate per 1000 women with at least one registered cervical sample should be interpreted as a regional annual cancer-detection rate scaled to cervical-sampling activity rather than as the individual risk of cancer among women undergoing cervical sampling in that year.

The absence of a measurable SCC decline also underlines the continuing importance of regular screening participation and timely investigation of symptoms. Screening cannot prevent every cervical cancer, particularly among women who do not attend screening, have incomplete follow-up, or develop lesions that are difficult to detect by cytology. Symptomatic women therefore require clinical assessment regardless of their previous screening history or the timing of their most recent cervical sample.

Recent migration may be relevant as a contextual factor for future studies of cervical screening and cancer occurrence in Northern Norway. Approximately 63,400 Ukrainians arrived in Norway between February 2022 and the end of 2023, with women comprising a substantial proportion of this population [38]. Cervical screening in Ukraine has largely been opportunistic rather than invitation based [39], and studies have reported low previous screening participation among Ukrainian refugee women [40]. Norwegian registry data have likewise shown lower screening participation among some immigrant groups, particularly women from Eastern Europe [41], while a recent meta-analysis reported a modestly increased pooled risk of cervical cancer among migrant women, with substantial heterogeneity between populations and studies [42]. However, the present dataset contained no information on country of birth, migration history, or screening undertaken outside Norway. Migration could therefore not be evaluated in relation to the observed SCC trend and should be regarded only as a contextual factor for future research.

### 4.5. Relevance of the Largely Unvaccinated Study Population

Women aged 40–69 years during 1998–2025 were born in 1985 or earlier and were therefore ineligible by birth cohort for either the school-based HPV vaccination programme, introduced for girls born in 1997 or later, or the catch-up programme for women born in 1991–1996 [16]. Although some women may have been vaccinated privately, the observed reduction in CIN3+ is unlikely to reflect a direct effect of programme-based vaccination. This distinguishes the study population from younger Norwegian cohorts, in which marked reductions in vaccine-targeted HPV prevalence and high vaccine effectiveness against CIN2+ have been documented following vaccination, while CIN3+ declined as vaccinated birth cohorts entered organised screening [16,43,44].

The study population provides useful information about background trends during the same period in which vaccinated cohorts entered screening. Reductions observed among younger vaccinated women must be interpreted against simultaneous changes in screening methods and diagnostic practice. The more modest decline in CIN3+ and the absence of a significant temporal trend in SCC detection among older, programme-unvaccinated women suggest that screening-related changes may account for part of the temporal development, whereas larger reductions in younger vaccine-eligible cohorts may additionally reflect vaccination. Direct comparisons would nevertheless require standardised age groups, calendar periods, screening histories, and analytical methods.

Individual vaccination status was unavailable, and privately funded HPV vaccination outside the national programmes cannot be excluded. However, given the age of the study population, the population-level impact of such vaccination is expected to have been minimal. Indirect herd protection from high vaccination coverage in younger birth cohorts may also have reduced HPV transmission, but this effect is likely to have been limited in women aged 40–69 years, who contribute less to ongoing oncogenic HPV circulation than younger age groups. Norwegian modelling indicates that the population-level effects of HPV vaccination accumulate over several decades and that vaccination, combined with HPV-based screening, could lead to cervical cancer elimination in Norway by 2039 [45]. Thus, vaccination is unlikely to have materially influenced the observed trends in this largely programme-unvaccinated population during most of the study period.

### 4.6. Strengths and Limitations

The principal strength of this study is the long observation period, covering the transition from cytology-based screening to HPV-based primary screening. UNN is the sole pathology provider for Troms and Finnmark, providing near-complete regional coverage of cervical cytology, HPV testing, biopsies, excisional specimens, and histological diagnoses. Standardised NORPAT codes enabled consistent identification of outcomes, and person-specific de-duplication ensured that each woman was counted once per calendar year according to her most severe diagnosis. Relating histological outcomes to the annual number of women with at least one registered cervical sample provides additional information beyond analyses based solely on absolute case counts.

The inclusion of CIN2+, CIN3+, and SCC as separate but nested endpoints was another strength. CIN2+ captures the overall burden of lesions generating assessment and treatment, CIN3+ provides a more specific measure of advanced precancer, and SCC represents invasive squamous disease. The contrasting trends across these endpoints provide more information than any single outcome considered in isolation.

Several limitations must be considered. First, this was an ecological, repeated cross-sectional study and did not follow individual women longitudinally across the entire period. A woman could contribute records in several calendar years, and the outcomes represent annual detection rather than first-ever diagnoses. The study cannot determine whether earlier detection and treatment of CIN2 in an individual woman prevented subsequent CIN3 or SCC.

Second, the denominator included women with at least one registered cervical sample obtained through organised screening, follow-up, or clinical indications. It did not represent a closed screening cohort or the entire female population. Changes in screening intervals, participation, indications for testing, repeat sampling, and the age distribution of women undergoing cervical sampling may therefore influence the annual rates. Population-based, age-standardised incidence rates would provide a complementary measure, particularly for SCC.

Third, the primary analyses were not age-standardised and were not adjusted for screening history, HPV status, hysterectomy status, previous treatment, socioeconomic characteristics, migration, or other potential confounders. However, additional analyses stratified by age (40–49, 50–59, and 60–69 years) showed increasing CIN2+ detection in all three strata and negative CIN3+ trends in all three strata, although the latter reached statistical significance only among women aged 40–49 years. Residual differences in age composition within these 10-year age groups may nevertheless remain. Individual HPV mRNA and HPV DNA results were not linked to the annual histological outcomes in the present analysis. The contribution of specific screening technologies could therefore not be separated from concurrent changes in clinical practice.

Fourth, histological classification may have changed over time. CIN2 is particularly susceptible to interobserver variation and changes in the use of ancillary testing, diagnostic thresholds, and secondary review. Although NORPAT coding provided a stable framework, it cannot eliminate grade migration or differences in diagnostic interpretation.

Fifth, the annual number of SCC cases was small, limiting statistical power and producing substantial year-to-year variation. Negative binomial regression accounted for varying annual denominators and potential overdispersion, but the overall temporal models still represented calendar year as a single continuous term and therefore summarised the average long-term direction of change. The additional predefined-period analyses allowed some assessment of non-linearity, although the gradual and overlapping changes in screening practice preclude attribution of differences to discrete interventions.

Finally, the study was conducted in a geographically large but sparsely populated region. Screening participation, access to healthcare, and referral practices may differ from those in more densely populated areas, which may limit generalisability to other regions or countries.

### 4.7. Implications and Future Research

The increasing CIN2+ detection rate and declining CIN3+ detection rate support continued evaluation of how HPV-based screening, molecular triage, cytology quality assurance, and histological diagnostic practice interact. Future analyses should link individual screening results, HPV genotypes, cytology, histology, treatment, and subsequent outcomes. Such linkage would make it possible to determine whether women identified earlier with CIN2 have a reduced subsequent risk of CIN3+ or cancer.

Longer follow-up is required to evaluate the effect of universal primary HPV screening on invasive cancer. National or multi-regional studies should use age-standardised population denominators and cancer registry data, while distinguishing organised screening from follow-up and symptom-driven testing. With longer follow-up and more granular information on screening modality and clinical management, future studies could use segmented regression, interrupted time-series, or joinpoint approaches to examine non-linear changes associated with specific programme transitions, although overlapping interventions will remain an analytical challenge.

Genotype-specific HPV mRNA testing warrants further evaluation as a complementary risk-stratification tool within HPV DNA-based screening. Norwegian studies indicate that mRNA positivity identifies a comparatively small subgroup with a high risk of CIN3+ and cervical cancer, whereas mRNA-negative results are associated with substantially lower risk [15,26,27,33]. Prospective comparative studies should determine whether mRNA-based triage can maintain adequate sensitivity for clinically important disease while reducing unnecessary colposcopies and biopsies.

## 5. Conclusions

In this regional repeated cross-sectional pathology study of women aged 40–69 years, CIN2+ detection increased over time, CIN3+ detection decreased, and no significant temporal trend was observed for cervical SCC. These patterns occurred during substantial changes in screening and diagnostic practice, but the independent contributions of HPV-based screening, local HPV mRNA-based quality assurance, changing screening intervals, diagnostic activity, histopathological practice, and changes in the annual sampling denominator cannot be separated. The reported rates represent regional pathology detection relative to cervical sampling activity and should not be interpreted as population incidence rates. The SCC findings should be interpreted cautiously because of the small number of cases and limited follow-up after full implementation of primary HPV screening.

## Figures and Tables

**Table 1:** Annual numbers of women with ≥1 cervical biopsy, CIN2+, CIN3+, and cervical SCC in women Aged 40–69 years in Troms and Finnmark, 1998–2025.

Year	Women with ≥1 Cervical Biopsy, *n*	CIN2+, *n*	CIN3+, *n*	SCC, *n*
1998	1170	133	80	13
1999	1034	97	67	5
2000	907	85	66	6
2001	1029	71	57	8
2002	1210	88	67	7
2003	1090	87	68	4
2004	1086	78	62	2
2005	1047	72	58	2
2006	991	62	50	4
2007	958	72	53	4
2008	930	93	73	2
2009	918	92	61	2
2010	940	70	46	3
2011	1050	102	50	4
2012	1062	103	44	6
2013	1220	130	68	7
2014	1377	125	53	5
2015	1500	143	69	3
2016	1380	153	62	3
2017	1354	142	68	7
2018	1363	146	64	13
2019	1266	123	51	7
2020	1061	123	52	3
2021	1194	143	41	5
2022	1137	138	48	8
2023	1262	136	34	3
2024	1179	118	43	5
2025	1166	124	41	2
Total	31,881	3049	1596	143

Abbreviations: ACIS, adenocarcinoma in situ; CIN2, cervical intraepithelial neoplasia grade 2; CIN3, cervical intraepithelial neoplasia grade 3; SCC, squamous cell carcinoma. CIN2+ includes CIN2, CIN3, ACIS, and SCC; CIN3+ includes CIN3, ACIS, and SCC. For biopsy activity, each woman was counted once per calendar year if she had at least one registered cervical biopsy. For histological endpoints, each woman was counted once per calendar year according to her most severe diagnosis. The total number of women with ≥1 cervical biopsy represents annual woman-records and not 31,881 unique individuals across the entire study period.

**Table 2:** Annual cervical-sampling activity, biopsy activity, and detection rates of CIN2+, CIN3+, and cervical SCC in women aged 40–69 years in Troms and Finnmark, 1998–2025.

Year	Women with ≥1 Registered Cervical Sample, *n*	Women with ≥1 Cervical Biopsy (per 1000)	CIN2+ Rate (per 1000)	CIN3+ Rate (per 1000)	SCC Rate (per 1000)
1998	12,062	97.0	11.03	6.63	1.08
1999	12,306	84.0	7.88	5.44	0.41
2000	11,500	78.9	7.39	5.74	0.52
2001	11,665	88.2	6.09	4.89	0.69
2002	13,409	90.2	6.56	5.00	0.52
2003	12,379	88.1	7.03	5.49	0.32
2004	12,124	89.6	6.43	5.11	0.16
2005	11,818	88.6	6.09	4.91	0.17
2006	12,063	82.2	5.14	4.14	0.33
2007	12,316	77.8	5.85	4.30	0.32
2008	12,060	77.1	7.71	6.05	0.17
2009	12,821	71.6	7.18	4.76	0.16
2010	14,191	66.2	4.93	3.24	0.21
2011	16,036	65.5	6.36	3.12	0.25
2012	13,562	78.3	7.59	3.24	0.44
2013	14,265	85.5	9.11	4.77	0.49
2014	15,427	89.3	8.10	3.44	0.32
2015	13,661	109.8	10.47	5.05	0.22
2016	13,012	106.1	11.76	4.76	0.23
2017	14,184	95.5	10.01	4.79	0.49
2018	13,123	103.9	11.13	4.88	0.99
2019	12,165	104.1	10.11	4.19	0.58
2020	11,924	89.0	10.32	4.36	0.25
2021	13,454	88.7	10.63	3.05	0.37
2022	11,030	103.1	12.51	4.35	0.73
2023	9716	129.9	14.00	3.50	0.31
2024	9631	122.4	12.25	4.46	0.52
2025	8964	130.1	13.83	4.57	0.22
Overall	350,868	90.9	8.69	4.55	0.41

Abbreviations: ACIS, adenocarcinoma in situ; CIN2, cervical intraepithelial neoplasia grade 2; CIN3, cervical intraepithelial neoplasia grade 3; SCC, squamous cell carcinoma. CIN2+ includes CIN2, CIN3, ACIS, and SCC; CIN3+ includes CIN3, ACIS, and SCC. The biopsy rate is the number of women with ≥1 registered cervical biopsy per 1000 women with at least one registered cervical sample in the corresponding calendar year. Detection rates are reported as cases per 1000 women with at least one registered cervical sample. Each woman was counted once per calendar year for the cervical-sampling denominator and once for biopsy activity if she had at least one registered cervical biopsy. For histological endpoints, each woman was counted once per calendar year according to her most severe diagnosis. The overall denominator and biopsy total represent sums of annual woman-records; the same woman may contribute in more than one calendar year.

**Table 3:** Detection of CIN2+, CIN3+, and cervical squamous cell carcinoma according to major periods of cervical screening practice among women aged 40–69 years in Troms and Finnmark, 1998–2025.

Screening Period	Women with Cervical Sample, *n*	CIN2+, *n* (per 1000)	CIN3+, *n* (per 1000)	SCC, *n* (per 1000)
1998–2005 a	97,263	711 (7.31)	525 (5.40)	47 (0.48)
2006–2011 b	79,487	491 (6.18)	333 (4.19)	19 (0.24)
2012–2015 c	56,915	501 (8.80)	234 (4.11)	21 (0.37)
2016–2020 d	64,408	687 (10.67)	297 (4.61)	33 (0.51)
2021–2025 e	52,795	659 (12.48)	207 (3.92)	23 (0.44)
Overall, 1998–2025	350,868	3049 (8.69)	1596 (4.55)	143 (0.41)

a Cytology only. b Cytology with 5-type HPV mRNA triage of ASC-US/LSIL. c Cytology with 14-type HPV DNA triage of ASC-US/LSIL. d Cytology with 3-type HPV mRNA co-testing. e Primary HPV DNA screening. Rates are calculated as the number of diagnoses per 1000 annual woman-records with at least one registered cervical sample within each screening period. CIN2+ includes CIN2, CIN3, adenocarcinoma in situ (ACIS), and squamous cell carcinoma (SCC); CIN3+ includes CIN3, ACIS, and SCC. Screening periods represent the predominant screening and triage practices at the University Hospital of North Norway and should not be interpreted as discrete interventions.

**Table 4:** Annual number of women with a registered cervical sample by age group, Troms and Finnmark, 1998–2025.

Year	40–49 Years	50–59 Years	60–69 Years	Total
1998	5622	4230	2210	12,062
1999	5615	4254	2437	12,306
2000	5189	4075	2236	11,500
2001	5313	4226	2126	11,665
2002	5591	4975	2843	13,409
2003	5401	4449	2529	12,379
2004	5313	4341	2470	12,124
2005	4909	4336	2573	11,818
2006	4935	4338	2790	12,063
2007	5133	4444	2739	12,316
2008	5043	4164	2853	12,060
2009	5214	4356	3251	12,821
2010	5703	4941	3547	14,191
2011	6341	5313	4382	16,036
2012	5454	4567	3541	13,562
2013	5784	4732	3749	14,265
2014	6172	5085	4170	15,427
2015	5593	4405	3663	13,661
2016	5250	4296	3466	13,012
2017	5648	4786	3750	14,184
2018	5037	4527	3559	13,123
2019	4682	4283	3200	12,165
2020	4398	4317	3209	11,924
2021	4887	4904	3663	13,454
2022	4056	4034	2940	11,030
2023	3661	3539	2516	9716
2024	3500	3584	2547	9631
2025	3285	3328	2351	8964
Total	142,729	122,829	85,310	350,868

Counts represent annual woman-records with at least one registered cervical sample. The same woman may contribute in more than one calendar year.

**Table 5:** Age-stratified annual detection rates of CIN2+ and CIN3+ per 1000 women with at least one registered cervical sample, Troms and Finnmark, 1998–2025.

**Year**	**CIN2+ 40–49**	**CIN2+ 50–59**	**CIN2+ 60–69**	**CIN3+ 40–49**	**CIN3+ 50–59**	**CIN3+ 60–69**
1998	13.34	8.75	9.50	7.83	5.20	6.33
1999	10.15	6.58	4.92	7.48	4.00	3.28
2000	10.02	5.64	4.47	9.06	3.19	2.68
2001	8.85	3.79	3.76	6.78	3.79	2.35
2002	9.30	4.82	4.22	6.44	4.02	3.87
2003	9.26	5.39	5.14	7.59	3.82	3.95
2004	10.54	2.53	4.45	8.85	1.84	2.83
2005	7.33	5.77	4.28	6.31	4.15	3.50
2006	7.70	3.69	2.87	6.28	2.77	2.51
2007	10.13	2.25	3.65	7.40	1.80	2.56
2008	10.91	6.24	4.21	8.72	4.80	3.15
2009	11.89	5.05	2.46	7.86	3.21	1.85
2010	8.59	1.62	3.67	5.26	1.01	3.10
2011	12.14	3.20	1.83	6.15	1.69	0.46
2012	12.83	4.16	3.95	6.05	0.88	1.98
2013	14.52	5.49	5.33	6.92	3.17	3.47
2014	12.96	5.90	3.60	5.18	2.75	1.68
2015	16.27	7.04	5.73	7.51	4.09	2.46
2016	17.90	8.38	6.64	7.81	1.86	3.75
2017	15.58	9.19	2.67	6.91	4.81	1.60
2018	16.48	8.84	6.46	7.54	3.31	3.09
2019	16.02	6.07	6.88	6.41	2.80	2.81
2020	15.23	7.41	7.48	6.14	2.78	4.05
2021	17.60	7.95	4.91	4.50	2.45	1.91
2022	17.01	11.40	7.82	6.66	3.72	2.04
2023	18.57	13.56	7.95	5.19	2.54	2.38
2024	18.00	9.49	8.24	6.86	3.07	3.14
2025	19.79	12.32	7.66	7.00	4.21	1.70

Rates are calculated as the number of CIN2+ or CIN3+ diagnoses per 1000 women with at least one registered cervical sample in the corresponding calendar year and age group. CIN2+ includes CIN2, CIN3, adenocarcinoma in situ, and squamous cell carcinoma. CIN3+ includes CIN3, adenocarcinoma in situ, and squamous cell carcinoma.

## Data Availability

The individual-level data supporting the findings of this study are not publicly available because they were derived from routine pathology records and are subject to privacy requirements and institutional data-governance restrictions. Aggregated data underlying the reported results may be made available by the corresponding author upon reasonable request and subject to approval by the University Hospital of North Norway.

## Abbreviations

The following abbreviations are used in this manuscript:

ACIS	Adenocarcinoma in situ
AI	Artificial intelligence
CI	Confidence interval
CIN1	Cervical intraepithelial neoplasia grade 1
CIN2	Cervical intraepithelial neoplasia grade 2
CIN2+	Cervical intraepithelial neoplasia grade 2 or worse
CIN3	Cervical intraepithelial neoplasia grade 3
CIN3+	Cervical intraepithelial neoplasia grade 3 or worse
DNA	Deoxyribonucleic acid
HPV	Human papillomavirus
LEEP	Loop electrosurgical excision procedure
LLETZ	Large loop excision of the transformation zone
mRNA	Messenger ribonucleic acid
NORPAT	Norwegian Pathology Code System
REK Nord	Regional Committee for Medical and Health Research Ethics, Northern Norway
SCC	Squamous cell carcinoma
SPSS	Statistical Package for the Social Sciences
UNN	University Hospital of North Norway
